# Molecular Profiling Predicts the Existence of Two Functionally Distinct Classes of Ovarian Cancer Stroma

**DOI:** 10.1155/2013/846387

**Published:** 2013-05-09

**Authors:** Loukia N. Lili, Lilya V. Matyunina, L. DeEtte Walker, Benedict B. Benigno, John F. McDonald

**Affiliations:** ^1^Integrated Cancer Research Center, School of Biology and Parker H. Petit Institute of Bioengineering and Bioscience, Georgia Institute of Technology, 315 Ferst Dr., Atlanta, GA 30332, USA; ^2^Ovarian Cancer Institute, 960 Johnson Ferry Road, Suite 130, Atlanta, GA 30342, USA

## Abstract

Although stromal cell signaling has been shown to play a significant role in the progression of many cancers, relatively little is known about its importance in modulating ovarian cancer development. The purpose of this study was to investigate the process of stroma activation in human ovarian cancer by molecular analysis of matched sets of cancer and surrounding stroma tissues. RNA microarray profiling of 45 tissue samples was carried out using the Affymetrix (U133 Plus 2.0) gene expression platform. Laser capture microdissection (LCM) was employed to isolate cancer cells from the tumors of ovarian cancer patients (Cepi) and matched sets of surrounding cancer stroma (CS). For controls, ovarian surface epithelial cells (OSE) were isolated from the normal (noncancerous) ovaries and normal stroma (NS). Hierarchical clustering of the microarray data resulted in clear separations between the OSE, Cepi, NS, and CS samples. Expression patterns of genes encoding signaling molecules and compatible receptors in the CS and Cepi samples indicate the existence of two subgroups of cancer stroma (CS) with different propensities to support tumor growth. Our results indicate that functionally significant variability exists among ovarian cancer patients in the ability of the microenvironment to modulate cancer development.

## 1. Introduction

The epithelial cells of the ovary interact with the cells of the surrounding microenvironment in order to regulate tissue homeostasis. Morphologically, the normal ovarian epithelial cells form a flat-to-cuboidal monolayer supported by a basement membrane. Cells located below this basement membrane are composed of various cell types collectively referred to as stromal cells. The most common types of stromal cells are fibroblasts, pericytes, endothelial cells, and various immune and inflammatory cells. Stromal and epithelial cells communicate through the secretion and binding of growth factors and other signaling molecules that promote reciprocal cellular responses appropriate for coordinated cell functions, for example, those required for the replication of ovarian surface epithelial cells following ovulation [1–3].

During cancer progression, genetic and epigenetic alterations lead to changes in the morphology and behavior of both epithelial and stromal cells by disrupting the tissue architecture and by interfering with signaling mechanisms. For example, signaling changes in a wide variety of developing cancer cells have been shown to result in the disruption of tissue homeostasis by inducing extracellular matrix (ECM) turnover, basement membrane disassociation, and increased stromal cell proliferation [1, 4].

Despite the well-documented role of stromal cell signaling in cancer progression, relatively few studies have been focused specifically on epithelial ovarian cancer-stromal interactions (EOC-SI). Previously reported studies on EOC-SI have focused on particular stromal components [5, 6], on specific cell lines [7], and/or employed in-house fabricated microarrays of limited scope [8]. We report here the results of a study of EOC-SI using high-throughput gene expression (microarray) analysis of normal ovarian surface epithelial cells and cells captured from normal stroma, cancer epithelia, and cancer stroma using laser capture microdissection (LCM). Our results reveal the existence of two categories of ovarian cancer stroma. Analysis of ligand-receptor patterns of gene expression indicates that one of these classes of cancer stroma may be more permissive and one more resistant to associated cancer cell growth.

## 2. Materials and Methods

### 2.1. Tissue Collection

Tissues were collected at Northside Hospital (Atlanta, GA, USA) under appropriate Institutional Review Board protocols. Following resection, the tumor tissues were placed in cryotubes and immediately (<1 minute) frozen in liquid nitrogen. Samples were transported on dry ice to Georgia Institute of Technology (Atlanta, GA, USA) and stored at −80°C. All tissues were examined, and diagnoses were made by a pathologist. The histopathology for each sample is listed in Table 1.

For each of the cancer tissue samples, 7 mm frozen sections were cut from samples embedded in cryomatrix (Shandon) and attached to uncharged microscope slides. Immediately following dehydration and staining (HistoGene, LCM Frozen Section Staining Kit, Arcturus), slides were processed in an Autopix (Arcturus) instrument for laser capture microdissection (LCM) of cancer epithelial cells (CEPI), cancer stroma (CS), and normal stroma (NS) using CapSure Macro-LCM Caps (Arcturus). Approximately 30,000 cells were collected from each of the samples. Normal ovarian surface epithelial (OSE) cells were also collected from normal ovaries at the time of surgery by light brushing using a Cytobrush Plus (Medscand), immediately stabilized in RNAlater (Ambion), and subsequently stored at −20°C. Microscopic examination of all collected cells was carried out to confirm the integrity and purity of the samples.

### 2.2. RNA Extraction and Amplification

PicoPure RNA Isolation Kit (Arcturus) protocols were followed for RNA extraction from the LCM cells on the Macro-LCM caps in 25 *μ*L of extraction buffer. Normal OSE cells were pelleted from RNAlater; RNA was isolated with Trizol (Invitrogen) and purified with the PicoPure RNA Isolation Kit. RNA quality was verified for all samples on the Bioanalyzer RNA Pico Chip (Agilent Technologies).

Total RNA from the above extractions was processed using the RiboAmp HS Kit (Arcturus) in conjunction with the IVT Labeling Kit from Affymetrix, to produce an amplified, biotin-labeled mRNA suitable for hybridizing to GeneChip Human Genome U133 Plus 2.0 Arrays (Affymetrix) following manufacturer's recommendations.

### 2.3. Microarray Data Analysis

We generated 45 individual gene expression profiles from 12 OSE brushings and 18 Cepi, 8 NS, and 7 CS patient samples isolated by laser capture microdissection (LCM). Affymetrix CEL files were processed using the Affymetrix Expression Console (EC) Software Version 1.1 with the default MAS5.0 probeset normalization algorithm. The expression values from the 12 OSE, 18 Cepi, 8 NS, and 7 CS samples were log_2_ transformed and then averaged for each probeset across each sample type. The microarray data were deposited in the Gene Expression Omnibus (GSE38666).

Probesets (genes) with nearly constant expression values (log_2_ normalized) across samples (SD < 1) were excluded from further consideration. Of the 54,675 probesets on the U133 Plus 2.0 chip, 42,698 were thus retained. A four-way ANOVA was subsequently employed to identify genes significantly differentially expressed (*P* ≤ 0.001) across the four sample groups (OSE, Cepi, NS, and CS). These 6,654 genes were employed in the initial clustering analysis.

A subsequent comparison among the CS samples (CS_1_ and CS_2_) alone was performed using a similar approach. Specifically, genes with nearly identical expression values (SD < 1) across CS_1_ and CS_2_ were discarded, and the remaining 38,972 genes were subjected to an unpaired *t*-test to identify those genes that were significantly differentially expressed between the CS_1_ and CS_2_ subgroups (*P* ≤ 0.001, 88 genes).

All heat maps were generated using the UPGMA (unweighted average) clustering method and the Euclidean distance similarity measure.

### 2.4. Ligand-Receptor Compatibility Analysis

For the ligand-receptor compatibility analysis, probesets associated with no or marginal expression across all 45 samples were discarded resulting in 5,865 differentially expressed genes. The presence or absence of the expression in samples was determined using the Affymetrix default MAS 5.0 decision algorithm. The MAS 5.0 algorithm uses Tukey's biweight estimator to provide a robust mean signal value and the Wilcoxon's rank test to calculate the significance of the signal or *P* value and detection call (present, marginal, or absent) for each probeset. The *P* values upon which the presence-absence calls for each ligand and receptor are based are presented in the appropriate Tables 1–5.

## 3. Results

### 3.1. Hierarchical Clustering Establishes Two Distinct Classes of Stroma among the Ovarian Cancer Patient Samples

**Figure 1:**
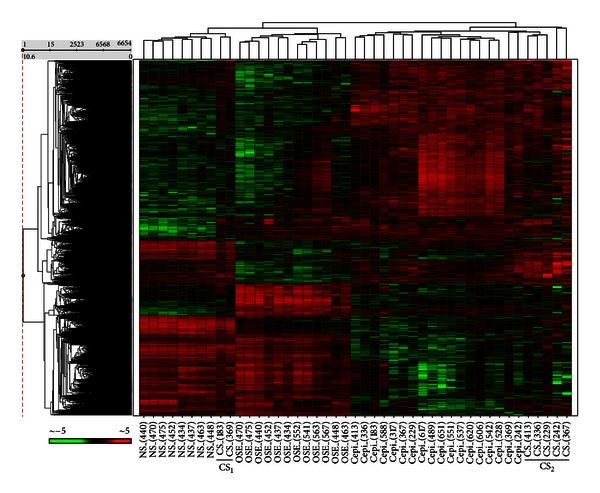
Hierarchical clustering of OSE, Cepi, NS, and CS expression profiles. The heat map was generated by *z*-score normalization of log_2_ expression values from Affymetrix HG U133 Plus 2.0. The results show that the OSE, Cepi, NS, and CS samples cluster into separate groups. The CS samples clustered into two distinct subgroups (CS_1_ and CS_2_).

Forty-five gene expression profiles were generated from 12 OSE brushings and 18 Cepi, 8 NS, and 7 CS patient samples isolated by laser capture microdissection (LCM). The relevant histopathologies of these 45 samples are listed in Table 1. Expression analysis yielded 6,654 differentially expressed probesets among the four sample types (ANOVA, *P* ≤ 0.001). Hierarchical clustering of these expression data resulted in clear separations between the OSE, Cepi, NS, and CS samples (Figure 1). Interestingly, the CS samples subdivided into two distinct groups. One (CS_1_) was more closely associated with the NS samples, and the other (CS_2_) was more closely associated with the Cepi samples.

One possibility is that the two subclasses of CS are simply a reflection of differential responses of stroma to molecular differences in the adjacent Cepi. If this were the case, we would expect to see a correlated substructure among the molecular profiles of the Cepi samples associated with the CS_1_ and CS_2_ subgroups. As shown in Figure 1, no such coordinated substructure pattern exists among the Cepi samples indicating that the two subclasses of CS are not merely a reflection of differential responses of the stroma to different Cepi subtypes.

As stated above, microscopic examination of LCM collected cells was carried out to validate the integrity of our samples. As a further confirmation, we conducted an additional computational analysis. In this analysis, probesets associated with no or marginal expression across all 45 samples were discarded resulting in 5,865 differentially expressed genes. If the reason for the presence of two classes of CS samples is that the CS_2_ class was a mixture of stroma and invasive Cepi cells, the gene expression levels in the putative mixed cancer stromal class (CS_2_) would be expected to lie within the range of the maximum and minimum expression levels of the NS and Cepi groups (i.e., avg(CS_2_) < Min and avg(CS_2_) > Max). Inconsistent with this prediction, we found that 2,342 or 40% (2,342/5,865) of the differentially expressed genes making up the CS_2_ class displayed values outside the predicted range of the mixed cell types. The fact that 60% of the expression values lie within the predicted range is not indicative of contamination but rather of the fact that not all genes are significantly overexpressed in the stroma relative to cancer samples. Collectively our microarray results are consistent with the microscopic examination in demonstrating the absence of infiltrating Cepi cells in the cancer stroma samples.

### 3.2. Gene Expression Patterns Are Consistent with the Existence of Ligand-Receptor Interactions between Cepi and CS

The significance of the presence of two distinct classes of ovarian cancer stroma may involve differential interaction between these stromal and the adjacent cancer cells. To explore this possibility, we first examined the expression levels of genes encoding signaling ligands and compatible receptors in the CS and Cepi datasets.

Two lists were established from the 5,865 differentially expressed probesets across the OSE, Cepi, NS, and CS samples. One list is comprised of all differentially expressed gene probes (note that each gene may be represented by multiple, nonoverlapping probes) encoding secreted ligands (ligand list) and the other of all expressed gene probes encoding surface receptors (receptor list) with documented binding affinity to the differentially expressed ligands (compatible ligands and receptors). The ligand list consists of 34 CS and 36 Cepi ligands while the receptor list is comprised of 20 Cepi and 21 CS receptors (Tables 2(a) and 2(b)).

We considered the expression of a ligand in CS (or Cepi) and its compatible receptor in Cepi (or CS) to be indicative of a potential CS-Cepi signaling interaction. Based on these criteria, we identified potential epithelial cancer-stroma signaling interactions (34 CS ligands and 20 Cepi receptors, see Table 2(a) and 36 Cepi ligands and 21 CS receptors, see Table 2(b)). Of these, there were 17 compatible pairs for both the CS ligands-Cepi receptors and Cepi ligands-CS receptors interactions (Table 3). Viewed from the perspective of individual genes (i.e., combining multiple probes of the same genes), there were 12 unique CS ligand-Cepi receptor pairs and 12 unique Cepi ligand-CS receptor pairs in our observed dataset (Table 4).

To determine if the observed coexpression of these 17 pairs of compatible ligands and receptors (probes) was greater than what would be expected by chance, we generated two lists. One list of observed data consisted of the expressed probes of the 17 CS ligands and 17 compatible Cepi receptors (Table 3(a)) and the other of the 17 expressed Cepi ligands and 17 CS receptors (Table 3(b)). A second list of random associations was generated using the same number of pairings as in the observed list (17 random pairs) and randomly selecting 17 pairs of ligands and receptors. One randomly selected CS (or Cepi) ligand from the pool of the 34 CS-(or 36 Cepi-) expressed ligands (Table 2(a)) was paired with one randomly selected Cepi (or CS) receptor from the pool of the 20 Cepi-(or 21 CS-) expressed receptors (Table 2(b)). These random associations were generated 100 times, and each time the number of biologically compatible ligand-receptor pairs arising by chance was counted. The number of biologically compatible interactions in the observed data (17) was then compared to the number of compatible interactions scored from the randomized associations using *z*-statistics. Two types of comparisons were performed, one for the pairs of CS ligands and Cepi receptors and another for the pairs of Cepi ligands and CS receptors. For both comparisons, the observed number of biologically compatible ligand-receptor pairs was significantly greater than what is expected by chance (CS ligands-Cepi receptors *z*-score = −4.68, *P* ≤ 0.0002; Cepi ligands-CS receptors *z*-score = −4.35, *P* ≤ 0.0002). Thus, the observed coexpression of pairs of compatible ligands and receptors is biologically significant.

### 3.3. Specific Ligand-Receptor Pairs between Cepi and CS Show Differential Gene Expression in the Two CS Classes

Of the 24 compatible pairs of ligand- and receptor-encoding genes listed in Table 4, most display similar expression patterns between CS_1_ and CS_2_. However, 6 of the ligand and receptor pairs display differential patterns of expression between the two groups of CS suggesting that CS_2_ may be a more conducive microenvironment for tumor growth (Table 5). For example, the FGF2 ligand, a documented inhibitor of tumor growth [9], is expressed in NS and in CS_1_ but not in CS_2_. Since a compatible receptor of this inhibitor (FGFR3) is expressed in Cepi, CS_2_ may be a more conducive microenvironment for tumor growth than CS_1_. The interleukin-7 (IL7) ligand has been previously implicated as an inducer of tumor growth in lymphoblastic leukemia [10], prostate cancer [11], breast cancer [12], and colorectal cancer [13]. IL7 is expressed in CS_2_ but not in CS_1_, again suggesting that CS_2_ may be a more conducive microenvironment for tumor growth than CS_1_.

The well-documented cancer-inducing ligands FGF1 and FGF9 [14–16] are both highly expressed in Cepi. The fact that the compatible FGFR3 receptor is expressed in CS_2_ but not in CS_1_ again suggests that CS_2_ is a more favorable microenvironment for ovarian cancer growth than CS_1_.

The *WNT* family of genes is involved in a variety of developmental processes, and aberrant expression of various members of *WNT* genes has been implicated in cancer [17]. For example, WNT7A is a ligand present in the extracellular matrix that participates in the sexual development of the Mullerian ducts [18]. Recent *in vivo *mouse studies suggest that WNT7A is an inducer of ovarian cancer growth [19]. Consistent with this interpretation, WNT7A has recently been identified as a potential early stage biomarker of human ovarian cancer [20]. The fact that WNT7A is expressed in CS_2_ but not in CS_1_ is also consistent with the hypothesis that CS_2_ may be a more conducive microenvironment for ovarian cancer growth than CS_1_.

A second member of the WNT family, WNT2B, is expressed in CS_1_ but not CS_2_ suggesting, contrary to what is presented above, that CS_1_ may be more permissive for cancer growth. However, the fact that WNT2B has been previously reported to be expressed in normal ovaries [21] coupled with our finding that it is also expressed in NS makes interpreting the significance of the dichotomy in WNT2B expression between CS_1_ and CS_2_ ambiguous.

## 4. Discussion

Cancer progression is a dynamic process involving cellular adaptation and survival that is, in part, driven by signaling interactions between participating cells. Many signaling interactions have been documented to take place between cancer epithelial cells and the surrounding stroma [22]. Early in tumor development, cancer cells produce growth factors that are believed to modulate or “activate” the surrounding stroma in order to convert the stroma into a supportive microenvironment for cancer growth [2, 14]. For example, it has been shown that growth factors secreted by macrophages can contribute to cancer progression and metastasis [23]. Other inflammatory cells such as lymphocytes, neutrophils, mast cells, T-regulatory cells, and platelets also have been shown to have the potential to support tumor progression by negatively regulating the anticancer host immune response [24–26]. Fibroblasts, the major component of the stroma, have been shown to be able to participate actively in the malignant progression of cancer by producing growth factors, various chemokines, and extra cellular matrix components that facilitate the production of endothelial cells and pericytes conducive to tumor growth [14, 27].

The purpose of this study was to investigate the process of stroma activation within the context of ovarian cancer. Toward this end, we conducted RNA microarray profiling of 45 tissue samples using the Affymetrix (U133 Plus2) gene expression platform. Laser capture microdissection (LCM) was used to isolate cancer cells from the tumors of 18 ovarian cancer patients (Cepi). For 7 of these patients, a matched set of surrounding cancer stroma (CS) was also collected. For controls, we isolated surface epithelial cells (OSE) from the normal (noncancerous) ovaries of 12 individuals including matched sets of samples of OSE and normal stroma (NS) from 8 of these patients.

Unsupervised hierarchical clustering of the microarray data resulted in the expected separation between the OSE and Cepi samples. Consistent with models of stromal activation, we also observed significant separation between the NS and CS samples. Somewhat unexpected, however, was our finding that the CS samples clustered into two distinct subgroups (CS_1_ and CS_2_).

Based on patterns of coexpression of ligand and receptor encoding genes, we determined that 6 biologically compatible pairs of ligands and receptors are differentially expressed between Cepi and the CS_1_ and CS_2_ cancer stroma. The patterns of differential expression between the compatible ligands and receptors are consistent with the hypothesis that CS_2_ may be a more conducive microenvironment for tumor growth (Table 5). For example, the expression of tumor promoting ligands in Cepi is always matched with the expression of compatible receptors in CS_2_ but not in CS_1_.

The fact that certain tumor microenvironments are capable of inhibiting tumor growth and/or development is well established. For example, macrophages can act as anticancer agents within the context of the innate immune response [28]. Likewise, fibroblasts, in some cellular contexts, have been shown to revert tumor cells to a normal, noncancerous phenotype [9, 29]. Normal ovarian stromal cells have been shown to significantly inhibit ovarian cancer cell growth when coinjected into nude mice [30].

The apparently innate anticancer properties of normal stroma are generally considered to be transient giving way to the “activation” of procancer growth signals induced by cancer cells as the tumors progress [1]. However, since the majority of the patients associated with the CS_1_ class of cancer stroma have, like the majority of the cancer patients included in our study, already progressed to advanced staged disease (Table 1), it is unlikely that the CS_1_ molecular profile represents a transient condition. Rather, our results suggest that variability exists among ovarian cancer patients with respect to the propensity of normal stroma to become activated.

## 5. Conclusions

An understanding of the potential clinical significance of the observed molecular dichotomy between ovarian cancer stroma is beyond the scope of this present study. However, it is relevant to note that all of the cancers associated with the putatively more permissive CS_2_ cancer stroma were classified as grade 3 while those associated with the putatively more resistant CS_1_ cancer stroma were classified as grade 2. The fact that no distinction was apparent between the molecular profiles of grade 2 and grade 3 Cepi samples (Figure 1) suggests that cancer grade may, at least in part, be determined by the relative permissiveness of the tumor microenvironment. Molecular profiling of larger numbers of matched sets of ovarian cancer and stroma samples will be required to adequately test this hypothesis. Nevertheless, the current results are consistent with the hypothesis that the microenvironment plays a significant role in ovarian cancer development and suggest that functionally significant variability may exist among ovarian cancer patients in the ability of the microenvironment to modulate cancer development.

## Figures and Tables

**Table 1:** Patient samples used in this study.

Patient ID	Age at time of surgery	Tissue for microarray	Histopathology	Stage	Grade
460	65	OSE	WNL (within normal limits)	N/A	N/A
552	41	OSE	WNL	N/A	N/A
563	66	OSE	WNL	N/A	N/A
567	78	OSE	WNL	N/A	N/A
434	41	OSE/NS	WNL	N/A	N/A
437	54	OSE/NS	WNL	N/A	N/A
440	50	OSE/NS	WNL	N/A	N/A
448	63	OSE/NS	WNL	N/A	N/A
452	51	OSE/NS	WNL	N/A	N/A
463	48	OSE/NS	WNL	N/A	N/A
470	44	OSE/NS	WNL	N/A	N/A
475	63	OSE/NS	WNL	N/A	N/A
317	59	Cepi	Serous adenocarcinoma	Ic	3
489	48	Cepi	Serous adenocarcinoma	IV	3
528	66	Cepi	Serous adenocarcinoma	IIIc	3
537	64	Cepi	Serous adenocarcinoma	IIIa	2
542	61	Cepi	Serous adenocarcinoma	IV	3
551	59	Cepi	Serous adenocarcinoma	IIIc/IV	3
588	71	Cepi	Serous adenocarcinoma	IIIc	2
606	54	Cepi	Serous adenocarcinoma	IIIa	3
617	64	Cepi	Serous adenocarcinoma	IIIc	2
620	62	Cepi	Serous adenocarcinoma	III/IV	3
651	46	Cepi	Serous adenocarcinoma	IIIb/IIIc	3
183	66	Cepi/CS	Serous adenocarcinoma	III	2
369	52	Cepi/CS	Serous adenocarcinoma	IIIc	2
229	58	Cepi/CS	Serous adenocarcinoma	IIIc	3
242	63	Cepi/CS	Serous adenocarcinoma	IIIb	3
336	63	Cepi/CS	Serous adenocarcinoma	Ic	3
367	56	Cepi/CS	Serous adenocarcinoma	II	3
413	49	Cepi/CS	Serous adenocarcinoma	IIb	3

**Table:** (a)

CS ligands		Cepi receptors	
Gene symbol	Probeset ID	Gene symbol	Probeset ID
***CXCL1	204470_at	****CXCR4	217028_at
*CXCL3	207850_at	**FGFR2	208228_s_at
***CXCL9	203915_at	***FGFR3	204379_s_at
***CXCL10	204533_at	**MET	203510_at
***CXCL11	210163_at	*TGFBR2	207334_s_at
***CXCL12	209687_at	***TGFBR2	208944_at
***CXCL12	203666_at	**TGFBR3	204731_at
***CXCL13	205242_at	***TGFBR3	226625_at
**CXCL16	223454_at	***PDGFRA	203131_at
CXCL17	226960_at	*PDGFRA	1554828_at
*FGF1	205117_at	***IL1R1	202948_at
*FGF2	204422_s_at	*IL1R1	215561_s_at
***FGF7	1554741_s_at	*IL1R2	205403_at
*FGF9	239178_at	**IL7R	226218_at
**FGF9	206404_at	**IL10RA	204912_at
***FGF13	205110_s_at	**FZD1	204451_at
*HGF	210997_at	**FZD2	210220_at
***IGF1	209540_at	**FZD7	203705_s_at
*IGF2	202409_at	***FZD7	203706_s_at
*TGFA	205016_at	**FZD10	219764_at
***TGFB2	209909_s_at		
*PDGFA	205463_s_at		
***PDGFD	219304_s_at		
*IL7	206693_at		
***IL15	205992_s_at		
**IL16	209828_s_at		
**IL17D	227401_at		
**IL18	206295_at		
**WNT2B	206458_s_at		
*WNT7A	210248_at		
***WNT5A	213425_at		
***VEGFA	210512_s_at		
*VEGFA	210513_s_at		
*VEGFA	211527_x_at		

**Table:** (b)

Cepi ligands		CS receptors	
Gene symbol	Probeset ID	Gene symbol	Probeset ID
***CXCL1	204470_at	****CXCR4	217028_at
**CXCL3	207850_at	**FGFR2	208228_s_at
***CXCL9	203915_at	*FGFR3	204379_s_at
***CXCL10	204533_at	IL12RB1	1552584_at
***CXCL11	210163_at	***IL1R1	202948_at
*CXCL12	203666_at	***TGFBR2	208944_at
*CXCL12	209687_at	***TGFBR3	204731_at
**CXCL13	205242_at	***TGFBR3	226625_at
**CXCL16	223454_at	***PDGFRA	203131_at
CXCL17	226960_at	**PDGFRA	215305_at
*FGF1	205117_at	***MET	203510_at
***FGF9	206404_at	**IL1R1	215561_s_at
***FGF9	239178_at	*IL1R2	205403_at
*FGF11	227271_at	**IL7R	226218_at
***FGF18	231382_at	**IL10RA	204912_at
*FGF18	211029_x_at	*IL21R	221658_s_at
*FGF18	206987_x_at	**FZD1	204451_at
**FGF18	214284_s_at	**FZD2	210220_at
**TGFA	205016_at	***FZD7	203705_s_at
**TGFB2	209909_s_at	**FZD7	203706_s_at
**PDGFA	205463_s_at	***FZD10	219764_at
***PDGFD	219304_s_at		
***IGF1	209540_at		
*IL7	206693_at		
*IL1B	39402_at		
***IL15	205992_s_at		
**IL18	206295_at		
**WNT2	205648_at		
**WNT2B	206458_s_at		
***WNT5A	213425_at		
*WNT7A	210248_at		
**WNT11	206737_at		
***VEGFA	210512_s_at		
***VEGFA	210513_s_at		
*VEGFA	211527_x_at		
**VEGFA	212171_x_at		

Significance of detection calls: *P ≤ 0.05, **P ≤ 0.005, and ***P ≤ 0.0005.

**Table:** (a)

CS ligands	Probesets	Compatible Cepi receptors	Probesets
***CXCL12	203666_at	***CXCR4	217028_at
***CXCL12	209687_at	***CXCR4	217028_at
*FGF1	205117_at	**FGFR2	208228_s_at
*FGF1	205117_at	*FGFR3	204379_s_at
*FGF2	204422_s_at	*FGFR3	204379_s_at
**FGF9	206404_at	*FGFR3	204379_s_at
*FGF9	239178_at	*FGFR3	204379_s_at
*HGF	210997_at	*MET	203510_at
*PDGFA	205463_s_at	*PDGFRA	1554828_at
*PDGFA	205463_s_at	***PDGFRA	203131_at
***TGFB2	209909_s_at	*TGFBR2	207334_s_at
***TGFB2	209909_s_at	***TGFBR2	208944_at
*WNT2	205648_at	*FZD2	210220_at
**WNT2B	206458_s_at	*FZD10	219764_at
*WNT7A	210248_at	*FZD7	203705_s_at
*WNT7A	210248_at	**FZD7	203706_s_at
*IL7	206693_at	**IL7R	226218_at

**Table:** (b)

Cepi ligands	Probesets	Compatible CS receptors	Probesets
*CXCL12	203666_at	***CXCR4	217028_at
*CXCL12	209687_at	***CXCR4	217028_at
*FGF1	205117_at	**FGFR2	208228_s_at
*FGF1	205117_at	*FGFR3	204379_s_at
*FGF9	206404_at	*FGFR3	204379_s_at
FGF9	239178_at	*FGFR3	204379_s_at
*PDGFA	205463_s_at	***PDGFRA	203131_at
*PDGFA	205463_s_at	*PDGFRA	215305_at
*TGFB2	209909_s_at	***TGFBR2	208944_at
*WNT2	205648_at	*FZD2	210220_at
*WNT2B	206458_s_at	***FZD10	219764_at
*WNT7A	210248_at	***FZD7	203705_s_at
*WNT7A	210248_at	**FZD7	203706_s_at
*IL1B	39402_at	***IL1R1	202948_at
*IL1B	39402_at	*IL1R2	205403_at
*IL1B	39402_at	*IL1R1	215561_s_at
*IL7	206693_at	*IL7R	226218_at

Significance of detection calls: *P ≤ 0.05, **P ≤ 0.005, and ***P ≤ 0.0005.

**Table 4:** The unique compatible ligands and receptors as potential interactions between the Cepi and the CS samples when multiple probes from Tables 3(a) and 3(b) are combined.

CS ligands	Compatible Cepi receptors	Cepi ligands	Compatible CS receptors
***CXCL12	***CXCR4	*CXCL12	***CXCR4
*FGF1	**FGFR2	*FGF1	**FGFR2
*FGF1	*FGFR3	*FGF1	*FGFR3
*FGF2	*FGFR3	**FGF9	*FGFR3
**FGF9	*FGFR3	**PDGFA	***PDGFRA
*HGF	*MET	**TGFB2	***TGFBR2
*PDGFA	***PDGFRA	**IL7	*IL7R
***TGFB2	***TGFBR2	**IL1B	*IL1R1
*IL7	**IL7R	**IL1B	*IL1R2
*WNT2	*FZD2	**WNT2	*FZD2
**WNT2B	*FZD10	**WNT2B	***FZD10
*WNT7A	**FZD7	**WNT7A	***FZD7

Significance of detection calls: *P ≤ 0.05, **P ≤ 0.005, and ***P ≤ 0.0005.

**Table 5:** The unique compatible ligands and receptors from Table 4 showing the expression pattern in NS, CS_1_, CS_2_, and Cepi. The 6 bold signals had the same expression in NS and CS_1 _but different expression between CS_1_ and CS_2_ despite the fact that their compatible signals in Cepi were always expressed.

NS	Ligands	CS_1_	CS_2_	Receptors	Cepi
+	CXCL12	+	+	CXCR4	+
−	FGF1	+	+	FGFR2	−
−	FGF1	+	+	FGFR3	+
**+**	**FGF2**	**+**	**−**	**FGFR3**	**+**
+	FGF9	+	+	FGFR3	+
−	HGF	+	+	MET	+
+	PDGFA	+	+	PDGFRA	+
+	TGFB2	+	+	TGFBR2	+
**−**	**IL7**	**−**	**+**	**IL7R**	**+**
−	WNT2	+	+	FZD2	+
**+**	**WNT2B**	**+**	**−**	**FZD2**	**+**
**−**	**WNT7A**	**−**	**+**	**FZD7**	**+**

NS	Receptors	CS_1_	CS_2_	Ligands	Cepi

+	CXCR4	+	+	CXCL12	+
+	FGFR2	+	+	FGF1	+
**−**	**FGFR3**	**−**	**+**	**FGF1**	**+**
**−**	**FGFR3**	**−**	**+**	**FGF9**	**+**
+	PDGFRA	+	+	PDGFA	+
+	TGBFR2	+	+	TGFB2	+
+	IL1R1	+	+	IL1B	+
−	IL1R2	+	−	IL1B	+
+	IL7R	+	+	IL7	+
+	FZD2	+	+	WNT2	+
+	FZD2	+	+	WNT2B	+
+	FZD7	+	+	WNT7A	+

Expression is denoted with “+” (i.e., there is at least one Affymetrix present call with detection *P* value ≤ 0.05) and nonexpression with “−” (i.e., there are no Affymetrix present calls in the samples with detection *P* value ≤ 0.05).
